# Nitrogen oxides as dopants for the detection of aromatic compounds with ion mobility spectrometry

**DOI:** 10.1007/s00216-017-0265-2

**Published:** 2017-03-03

**Authors:** Urszula Gaik, Mika Sillanpää, Zygfryd Witkiewicz, Jarosław Puton

**Affiliations:** 1 Institute of Chemistry, Military University of Technology, Kaliskiego 2, 00-908 Warsaw, Poland; 2 School of Engineering Science, Laboratory of Green Chemistry, Lappeenranta University of Technology, Sammonkatu 12, 50130 Mikkeli, Finland

**Keywords:** Ion mobility spectrometry, Dopants, Aromatic compounds, Nitrogen oxides

## Abstract

Limits of detection (LODs) in ion mobility spectrometry (IMS) strictly depend on ionization of the analyte. Especially challenging is ionization of compounds with relatively low proton affinity (PA) such as aromatic compounds. To change the course of ion-molecule reactions and enhance the performance of the IMS spectrometer, substances called dopants are introduced into the carrier gas. In this work, we present the results of studies of detection using nitrogen oxides (NO_x_) dopants. Three aromatic compounds, benzene, toluene, toluene diisocyanate and, for comparison, two compounds with high PA, dimethyl methylphosphonate (DMMP) and triethyl phosphate (TEP), were selected as analytes. The influence of water vapour on these analyses was also studied. Experiments were carried out with a generator of gas mixtures that allowed for the simultaneous introduction of three substances into the carrier gas. The experiments showed that the use of NO_x_ dopants significantly decreases LODs for aromatic compounds and does not affect the detection of compounds with high PA. The water vapour significantly disturbs the detection of aromatic compounds; however, doping with NO_x_ allows to reduce the effect of humidity.

**Graphical Abstract:**
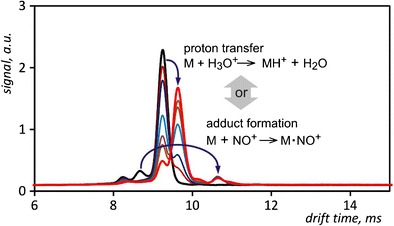
Two possible ionization mechanisms of aromatic compounds in ion mobility spectrometry: proton transfer reaction and adduct formation

Two possible ionization mechanisms of aromatic compounds in ion mobility spectrometry: proton transfer reaction and adduct formation

## Introduction

Ion mobility spectrometry (IMS) is a fast, simple and sensitive analytical technique used for the investigation of gaseous samples. The principle of this method is ions separation based on differences in their movements in the electric field [1, 2]. The range of applications of IMS is very wide [2–12] thanks to its many advantages such as a short analysis time, accuracy, low concentrations detectability, low costs of use or the possibility of performing real-time analysis [13] without the necessity of samples transportation to the laboratory.

IMS method sensitivity and limit of detection (LOD) are strictly related to analyte ions generation taking place in the reaction region of the spectrometer. Ionization processes depend on the composition of drift gas, temperature and the construction of a reaction region. The introduction of some substances, called dopants, to the gases flowing through the detector allows ion-molecule reactions occurring in the spectrometer to be controlled with high effectiveness [14, 15]. Dopant molecules form so-called alternative reactant ions, which interact with the analyte in a different way than the ions present in the pure carrier gas. Choosing a proper dopant allows for the generation of more stable ions containing the analyte molecule [16] or an increase in selectivity by shifting peaks in the drift time spectrum [17]. The most well-known use of dopants consists of inhibiting the ionization of substances which disturb the analyte detection in positive mode of IMS. For this purpose, ketone dopants, which are compounds of relatively high proton affinity (PA), are used very often [10, 18]. The reactant ions formed by these dopants molecules do not ionize interfering compounds molecules of low PA values. Therefore, better selectivity is accomplished by the significant elimination of cross-sensitivity effects. There is also a quantitative aspect of the use of dopants. Their presence may affect the detection efficiency and LOD. It was stated, however, that in the detection of organophosphorus compounds (OPC) using ketones as dopants, the detection sensitivity does not depend on the presence and concentration of the dopant [19, 20].

The detection of aromatic compounds like benzene, toluene or xylene (BTX) in the low concentration range is a very important analytical problem. This kind of aromatic compounds is commonly used in many branches of industry and is a serious threat to human health. The application of IMS in the effective detection of these substances is difficult, especially in cases where atmospheric pressure chemical ionization (APCI) is used to ionize the analyte molecules. The relatively low PA of the substances is considered the main reason for the low effectiveness of the detection of aromatics in positive mode of IMS [21]. Therefore, there have been attempts to apply detectors with UV ionisation sources [22, 23], where analyte ions are generated in the photoionization process. For some aromatic compounds, it is also possible to carry out measurements in negative mode of IMS. One of them is toluene diisocyanate (TDI) [24, 25], which is highly toxic and used in the production of polyurethane foams.

The lack of way to effectively ionize the aromatic compounds by proton transfer reactions does not entirely limit the possibility of generating stable positive ions for such analytes. Recently, it was demonstrated that the efficient ionization of aromatic compounds occurs in IMS detectors equipped with corona discharge (CD) ionization sources [26, 27]. Types of positive reactant ions from CD ionization sources are similar to those generated by radioactive sources. Nevertheless, the amounts of specific ions are definitely different. The reason for this is the generation of nitrogen oxides (NO_x_) between electrodes of the corona discharge ionization source [28]. The nitrogen monoxide (NO) reacting with products of air ionization is transformed to nitrosonium ion via the following reactions [27]:

1 $$ {{\mathrm{N}}_2}^{+} + \mathrm{N}\mathrm{O}\to \mathrm{N}{\mathrm{O}}^{+} + {\mathrm{N}}_2 $$

2 $$ {{\mathrm{O}}_2}^{+} + \mathrm{N}\mathrm{O}\to \mathrm{N}{\mathrm{O}}^{+} + {\mathrm{O}}_2 $$

Nitrogen monoxide is present in trace amounts in air or nitrogen used as drift gases. A small peak representing the signal generated by NO^+^ ions is therefore also observed in the drift time spectra obtained from measurements carried out with radioactive ionization IMS detectors. Due to the conditions in reaction regions of the IMS detectors, ions produced via reactions (1) and (2) are hydrated and form clusters with the chemical formula NO^+^(H_2_O)_n_. In corona discharge detectors, these ions might be dominant reactant ions, thus providing the opportunity for ionization through mechanisms other than proton transfer [29]. If the analyte molecule’s ionization energy (IE) is lower than 9.26 eV (the IE of nitrogen monoxide), then the charge transfer is possible:

3 $$ \mathrm{M} + \mathrm{N}{\mathrm{O}}^{+}\to {\mathrm{M}}^{+} + \mathrm{N}\mathrm{O} $$

Sufficiently low IE characterizes many organic analytes, among them are most of the aromatic compounds [30]. However, if the ionization energy of the analyte is higher than that of nitrogen monoxide, then sample ions may be produced by forming adducts (Eq. 4) or by hydride abstraction (Eq. 5):

4 $$ \mathrm{M} + \mathrm{N}{\mathrm{O}}^{+}\to {\left(\mathrm{M}\cdotp \mathrm{N}\mathrm{O}\right)}^{+} $$

5 $$ \mathrm{M} + \mathrm{N}{\mathrm{O}}^{+}\to {\left(\mathrm{M}\hbox{-} \mathrm{H}\right)}^{+} + \mathrm{H}\mathrm{N}\mathrm{O} $$

The application of NO^+^ ions allows the ionization of many kinds of analytes; the method of ionization, however, depends on the properties of the molecules.

The CD ionization source employed in IMS detectors may play a dual role. First, as for other ionization constructions, it produces an electric charge which is used to sample ions production and second, the nitrogen monoxide generated might be applied as a reaction gas with a beneficial effect on the sensitivity and selectivity of detection. Darzi and Tabrizchi [31] proposed the use of the chamber with the CD electrodes in the gas line before the inlet to the detector. This construction was solely designed for the efficient production of nitrogen monoxide. The advantage of such solution is the possibility of introducing the dopant without attaching gas cylinders or any other sources of NO. Moreover, it allows the dopant introduction to be switched on and off quickly. The application of CD sources as a nitrogen monoxide generator also has some limitations. It is not possible to produce stable and controlled levels of dopant concentrations in the drift gas. Attempts to add nitric oxide from gas cylinders into the carrier gas were carried out at an early stage of research on IMS [32]. It has been found that for the ionization of *n*-octane and 2-chloronitrobenzene, the reactivity of NO^+^ ions is higher than that of hydronium ions. Investigations of influence of nitrogen monoxide concentrations on the detection of 2,4-lutidine, di-tert-butyl-pyridine (DTBP) and dimethyl methylphosphonate (DMMP) were carried out by Eiceman et al. [33]. NO was added to the drift gas from the gas cylinder and an IMS detector was coupled with a gas chromatograph and mass spectrometer. The most crucial result of this research was the demonstration that there are several mechanisms of ionization when NO^+^ is used.

The purpose of this research is to investigate quantitative dependencies of the effect of nitrogen oxides on the detection of three chosen aromatic compounds. The analysis of two compounds of high PA (DMMP and triethyl phosphate (TEP)) as a comparison was also carried out. The measurements were conducted using a spectrometer equipped with a radioactive ionization source. Nitrogen oxides used as dopants were introduced to the system from gas cylinders. This provided sufficient accuracy for the quantitative determination of NO and NO_2_ concentrations.

## Experimental

### Gas generator and IMS detector

**Fig. 1:**
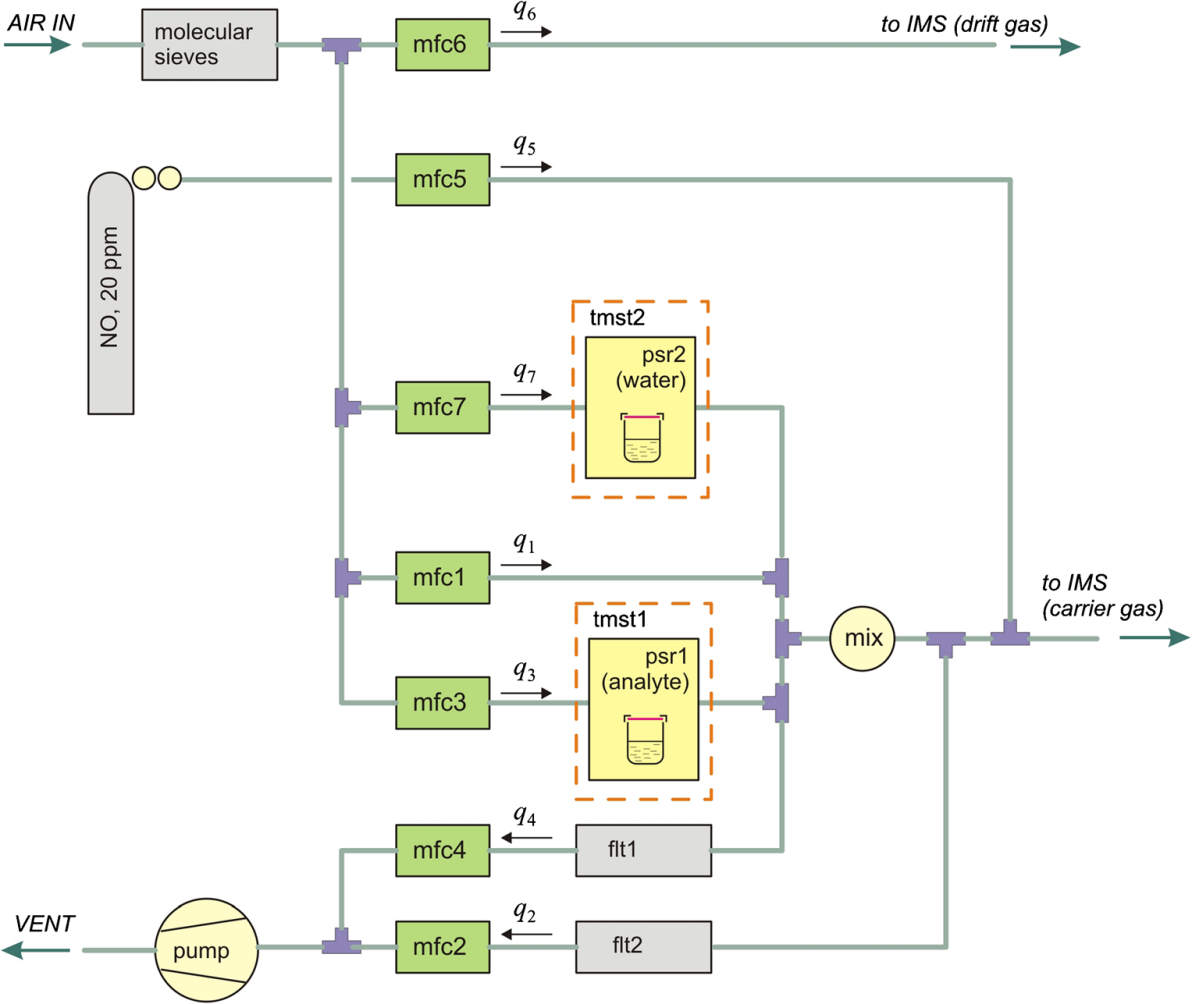
Scheme of the pneumatic system for the generation of gas mixtures. Abbreviations: *flt*—filter, *mfc*—mass flow controller, *tmst*—thermostatic container, *psr*—permeation source, *mix*—flow mixer

The measuring system applied in our research was adapted to produce gas mixtures containing two components, i.e. analyte and nitrogen monoxide or dioxide as a dopant. Moreover, in some of the measurements, the water vapour was also added. The scheme of the gas generator is presented in Fig. 1.

The analyte vapours were generated with permeation standards placed in the thermostatic container tmst1. The standards consisted of glass vials sealed with a polymer membrane. The material and the thickness of the membrane were chosen carefully to provide source emission, allowing measurements to be carried out in a dynamic range of detector response. The gas containing analyte vapours was mixed with additional stream of pure air in a system of single dilution. Gas flows were monitored using 7 mass flow controllers (mfc) 5850 series (Brooks Instruments). Both nitrogen monoxide and dioxide were introduced from gas cylinders. NO was added through the mass flow controller mfc5. The NO_2_ concentration in the gas cylinder was too high, therefore an additional dilution was necessary. Water vapour was added to the gas from an open vial placed in the thermostatic container tmst2. The emissions of an analyte as well as water vapour were determined based on weight loss of the sources. The concentrations of all components of the carrier gas were calculated using emission values and gas flow balances.

Parameters of the IMSD-B detector used for this research and a schematic of its reaction section are presented in [20]. It is a precise instrument dedicated for laboratory purposes. The device is equipped with radioactive ionization source ^63^Ni (300 MBq) and 6.1 cm long drift region.

The measurements were carried out at several different temperatures of the IMS detector. Toluene, benzene and DMMP detections doped with NO were conducted at 80 °C, and toluene detection doped with NO_2_ at 120 and 80 °C. Measurements of TDI with NO admixture were performed at 60, 90 and 120 °C. TEP with NO_2_ introduced to the carrier gas was tested at 80 °C. Detections of DMMP and toluene without dopants were carried out at 60 °C.

### Gases and chemicals

In all investigations, the carrier and drift gas was air purified and dried with molecular sieves 13× (Alfa Aesar) placed in a 2-L volume container. Analytes, i.e. benzene and toluene (Chempur), TDI and TEP (Sigma Aldrich), and DMMP (Alfa Aesar) were at least of analytical purity. Gas mixtures containing nitrogen monoxide (20 ppm) and nitrogen dioxide (500 ppm) were obtained from Air Products.

## Results and discussion

**Fig. 2:**
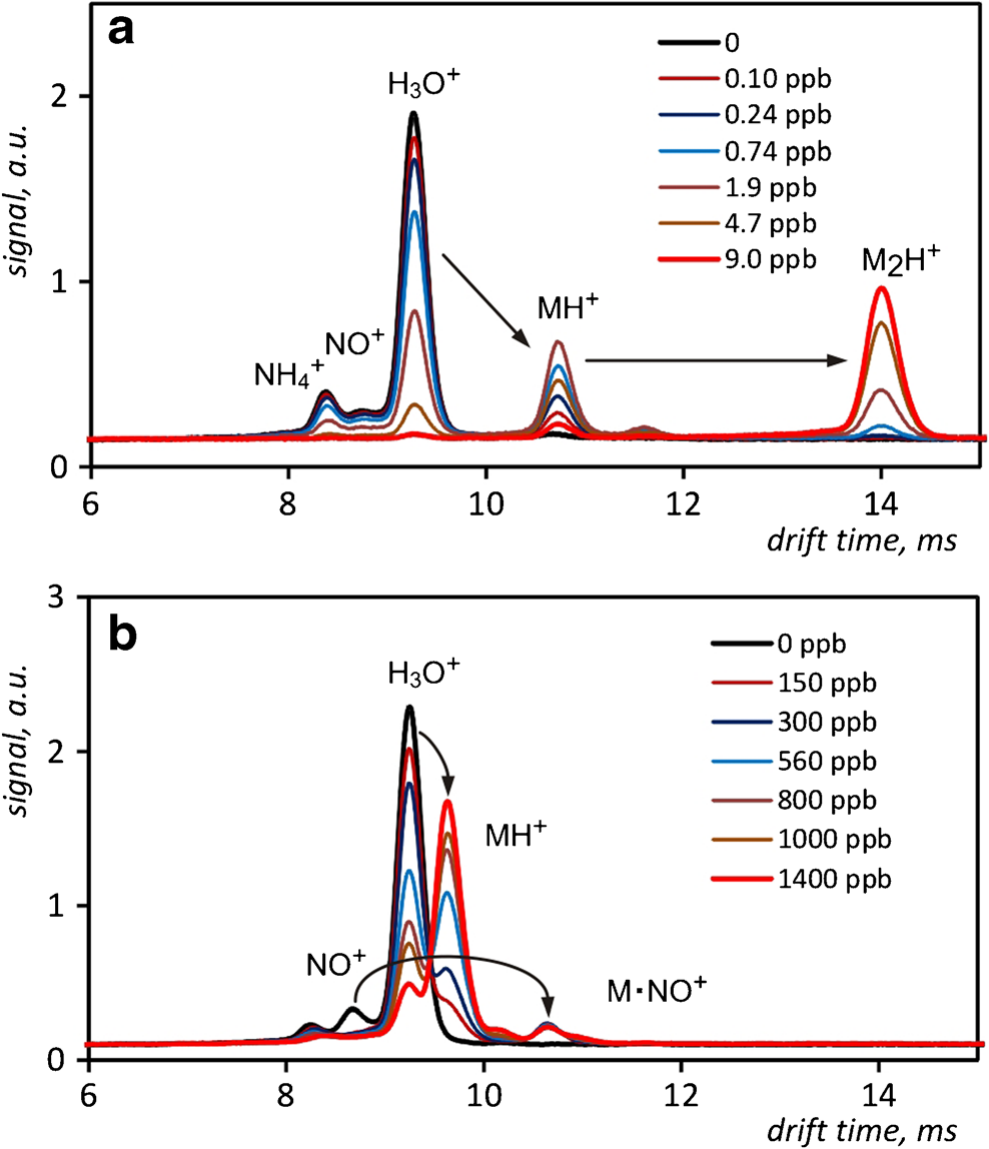
Drift time spectra of DMMP (**a**) and toluene (**b**) measured for the carrier gas without the dopant at 60 °C

There are three kinds of reactant ions in the positive mode of IMS (see Fig. 2a). The peak of greatest intensity observed in the drift time spectrum is generated by (H_3_O^+^)H_2_O_n_ ions. In much smaller amounts, there are also NH_4_^+^ and NO^+^ ions present. Usually, they are also hydrated. Under normal conditions in the IMS detector, all of the mentioned ions are hydrated. Introducing substances with high PA to the carrier gas causes the creation of ion products containing one or two molecules of the analyte. All three kinds of reactant ions participate in the ionization process, which results in an even decrease of peaks amplitudes with an increase of the analyte concentration. A typical example of the drift time spectrum showing this scheme of ionization was recorded for the detection of DMMP (Fig. 2a). The investigation of many aromatic compounds has shown that these chemicals behave differently. As an example, the results registered for increasing concentration of toluene can be shown (Fig. 2b). Decline of the hydronium reactant ion peak can be clearly observed only for concentrations higher than 100 ppm. Simultaneously, those concentration levels of toluene result in the complete disappearance of the NO^+^ ions peak. Thus, it can be concluded that the ionization of toluene molecules with nitrosonium ions is much more effective than for hydronium ions. The purpose of further research was to determine the quantitative characteristics of the detection of chosen analytes in the presence of nitrogen monoxide and nitrogen dioxide added intentionally to the carrier gas.

**Fig. 3:**
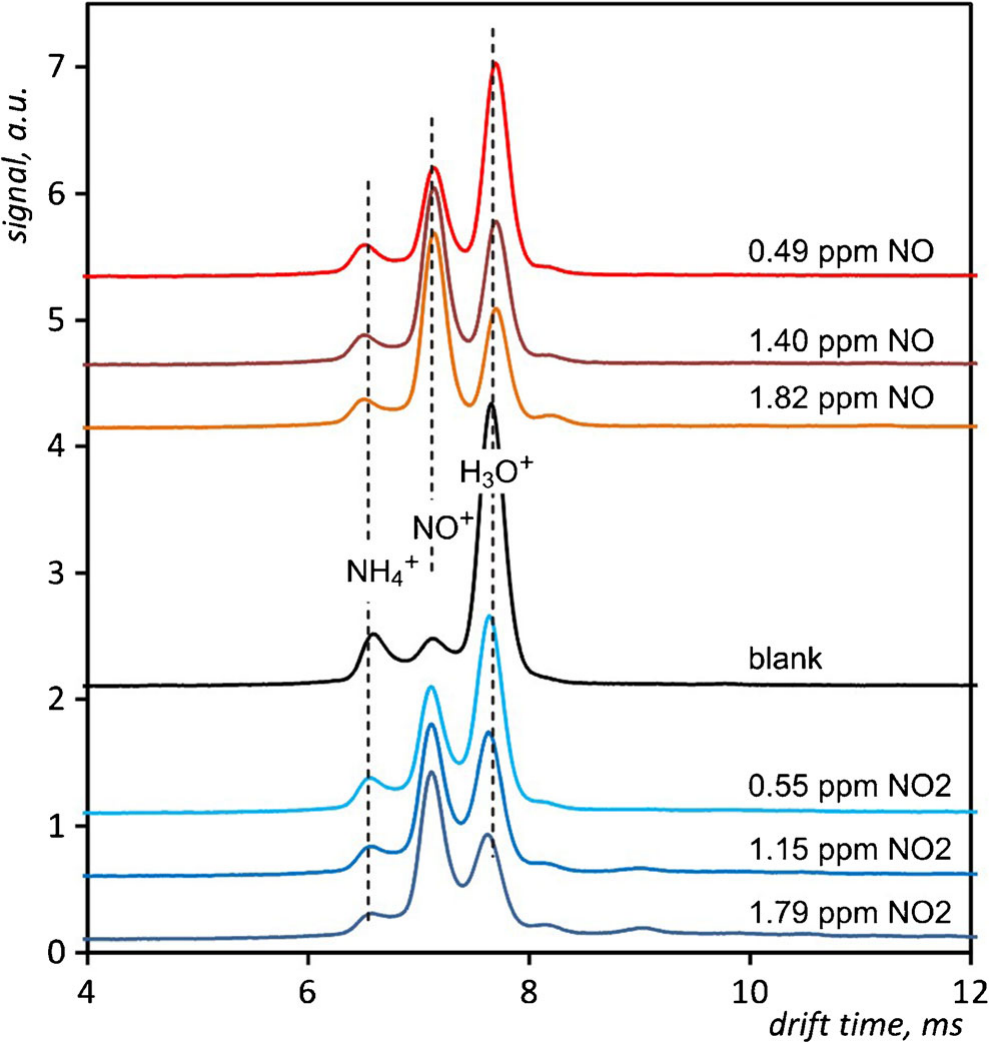
Drift time spectra of NO and NO_2_ registered for different concentrations at 120 °C

Drift time spectra measured for different concentrations of NO and NO_2_ are shown in Fig. 3. Spectra obtained for both dopants appear very similar. Minor differences are related to the ammonium ions peak. The presence of a nitrogen monoxide and nitrogen dioxide admixture decreases the height of the NH_4_^+^ peak.

**Fig. 4:**
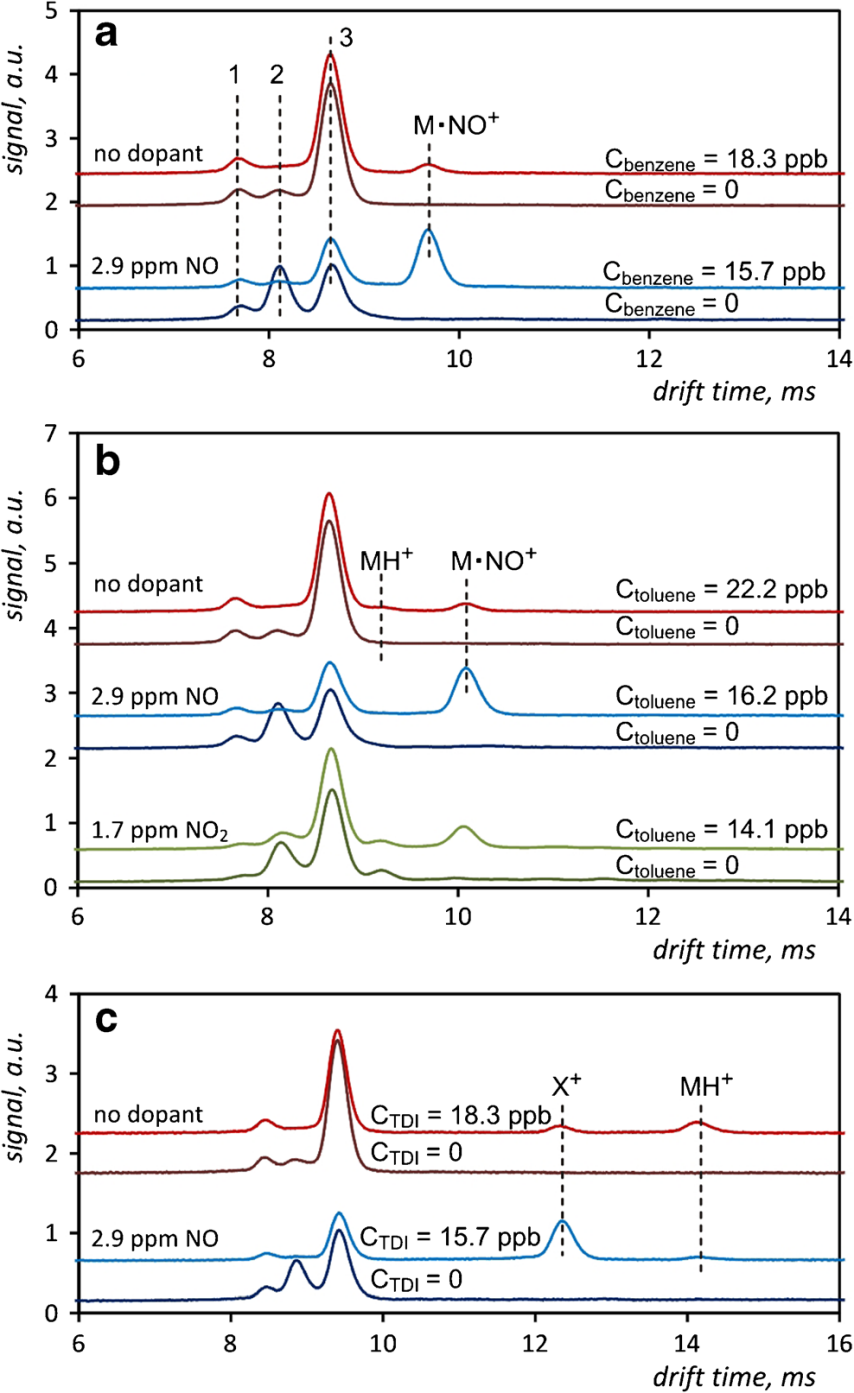
Drift time spectra of benzene measured in the presence of NO at 80 °C (**a**), toluene measured in the presence of NO and NO_2_ at 80 °C (**b**), and TDI measured in the presence of NO at 60 °C (**c**). Peaks marked 1, 2 and 3 in the panel **a** correspond to (NH_4_^+^)H_2_O_n_, (NO^+^)H_2_O_n_ and (H_3_O^+^)H_2_O_n_ ions, respectively

Research on the detection of toluene, benzene, TDI, DMMP and TEP using nitrogen oxides as the carrier gas dopants in the positive mode of IMS was conducted. The influence of NO and NO_2_ admixtures on the drift time spectra recorded for toluene, benzene and TDI is shown in Fig. 4. In the measurements carried out without dopants, ions generated as a result of interaction with NO^+^ are observed for all analytes. Except for the case of benzene, the peaks of the analytes ionized using hydronium ions are also visible. After the introduction of dopants, the peaks corresponding to the analyte ions produced by alternative reactant ions are significantly higher than those obtained with pure carrier gas. In the case of toluene and benzene, these peaks are shifted towards higher drift times compared to the ion peaks generated from hydronium ions. This may indicate that the corresponding analyte ions are characterized by a greater mass and collision cross-section. Therefore, it is possible that this type of ionization of analytes occurs through the reaction of adduct formation between the analyte molecule and the dopant ion. The described shift causes a complete separation of the peaks derived from the reaction of hydronium ions and ions of the analyte. This allows more accurate determinations of the tested substances to be conducted and, in the case of benzene, its detection to be performed because the values of the mobilities of hydronium ions and the benzene ions generated by them are very similar. Furthermore, because of the low proton affinity of this analyte, its ionization by hydronium reactant ions is very inefficient. Studies of toluene detection using nitrogen dioxide introduced to the carrier gas were also carried out. The influences of two admixtures are compared in Fig. 4b. It is interesting that the position of the peaks corresponding to the ions containing toluene molecules is the same for both dopants.

Ionization of TDI using nitrogen oxides is different from previous cases and the created peak is shifted towards the shorter drift times. This means that the product ions exhibit higher mobility than the ions produced using (H_3_O^+^)H_2_O_n_ ions, which generally also indicates a lower collision cross-section and/or weight of such ions. The ionization mechanism in this case probably differs from that for benzene and toluene. Two kinds of product ions are observed in the drift time spectra measured for TDI. The ions of lower mobility, with the drift time of 14.2 ms, are dominant for ionization without a NO dopant. Thus, it can be assumed that the fragmentation of a compound occurs when detection with IMS is carried out using nitrogen oxides as the carrier gas dopants.

**Fig. 5:**
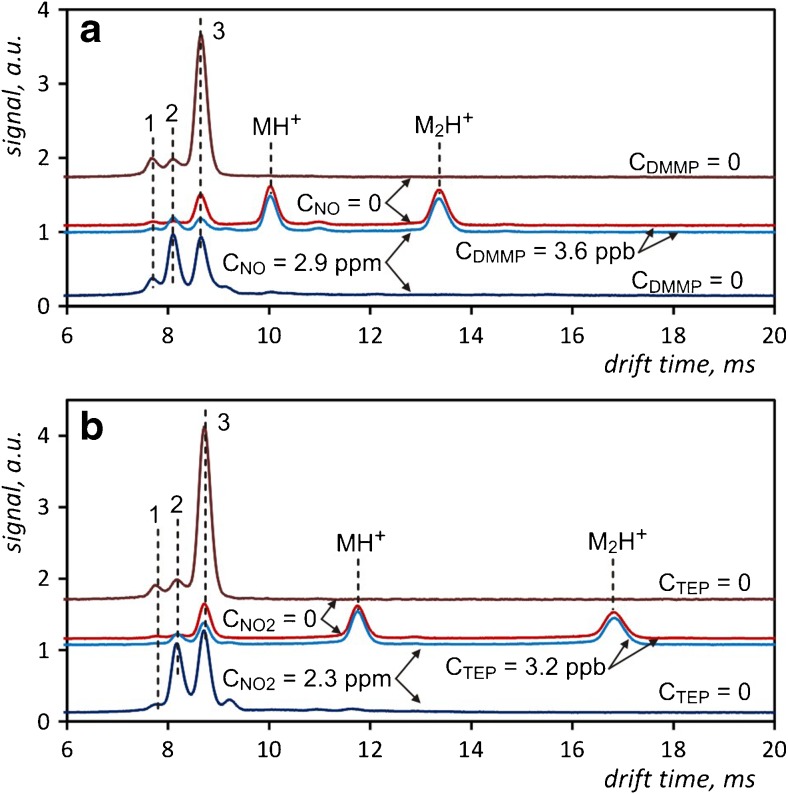
Drift time spectra of DMMP measured in the presence of NO (**a**), and TEP measured in the presence of NO_2_ (**b**) at 80 °C. Peaks marked 1, 2 and 3 in the panel **a** correspond to (NH_4_^+^)H_2_O_n_, (NO^+^)H_2_O_n_ and (H_3_O^+^)H_2_O_n_ ions, respectively

The influence of nitrogen oxides as dopants on the detection of compounds with high proton affinity, such as DMMP or TEP, which are easily ionized by standard reactant ions, was also investigated. DMMP detection was conducted using nitrogen oxide, and TEP using nitrogen dioxide. The results of these studies are shown in Fig. 5a, b, respectively. The drift time spectra are presented in such a manner that allows to show the set of peaks with the same mobilities and intensities to be shown which are observed irrespective of the dopant presence in the carrier gas. Therefore, it can be concluded that the efficiency of ionization and thus the detection of these analytes is not affected by the use of nitrogen oxides dopants. The results of research conducted by Eiceman et al. [33] using IMS coupled with mass spectrometry (IMS/MS) confirmed that both the analyte monomer and dimer ions have the same mass, regardless of which reactant ions were used (NO^+^ or (H_3_O^+^)H_2_O_n_). It is very interesting that the same analyte ions are obtained for different reaction mechanisms available for ionization by these two kinds of reactant ions. A similar phenomenon has already been observed for DMMP and TEP detected in the presence of ketone dopants [20]. However, this only concerned the conservation of dimer ions peaks intensities, while the monomer ions peaks were different depending on whether ketone dopants or pure carrier gas were used. In the case of NO_x_ doping, the peaks of both the monomer and the dimer ions of the analyte are the same.

**Fig. 6:**
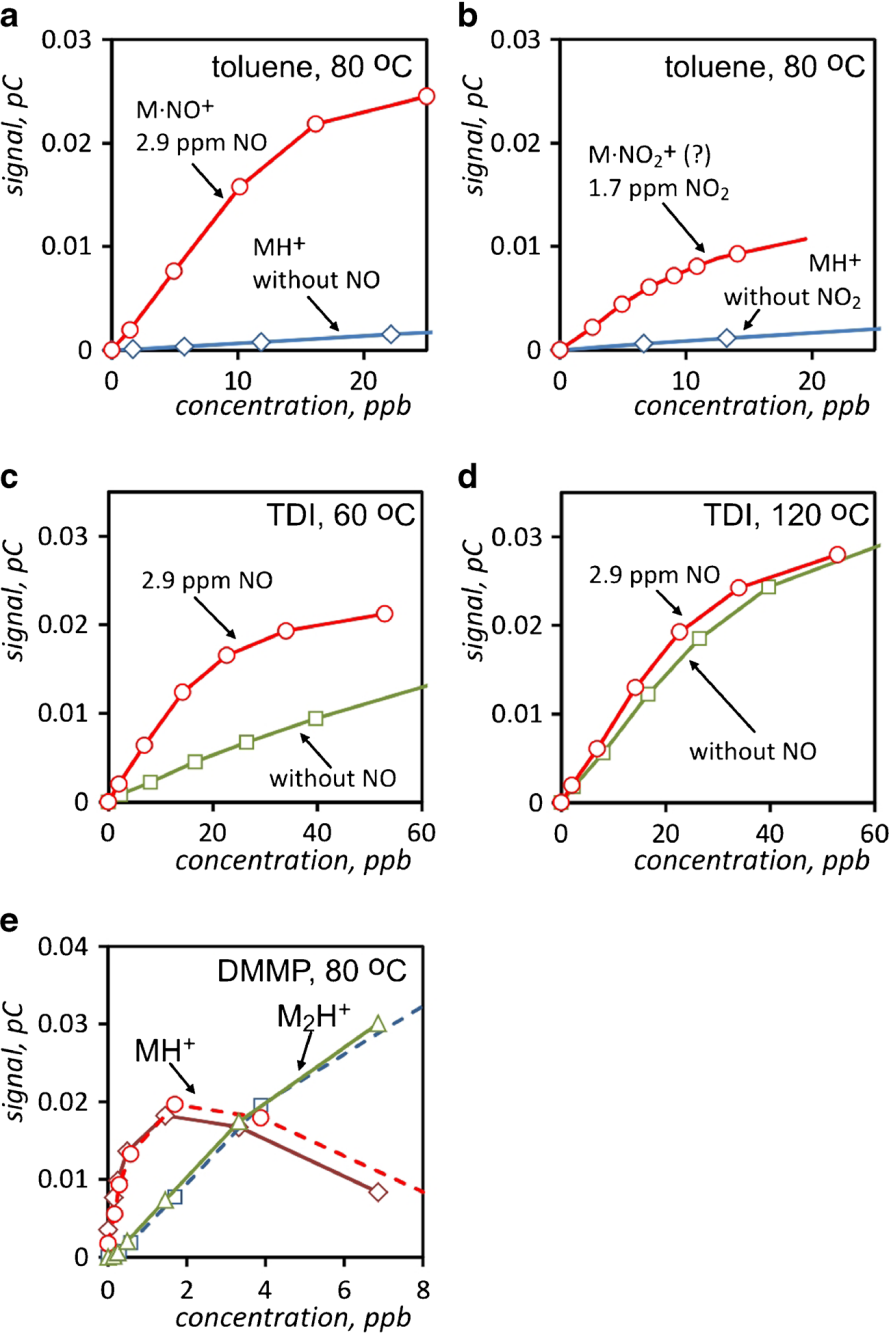
Calibration dependencies for toluene measured at 80 °C in the presence of NO (**a**) and NO_2_ (**b**), TDI measured in the presence of NO at 60 °C (**c**) and 120 °C (**d**) and DMMP measured at 80 °C in the presence of NO. *Dashed lines* in **e** are for pure air and solid for doped carrier gas

Quantitative assessment of the effectiveness of doping with nitrogen oxides is possible by comparing the calibration curves. Selected calibration curves are presented in Fig. 6. In general, calibration dependencies for IMS are non-linear. The limited linear dynamic range of the IMS detectors can cause the difficulties in quantitative analyses [34]. In the case of toluene, the signal from MH^+^ monomer ions, generated by hydronium ions, is small over the entire range of analyte concentrations. In contrast, the signal from toluene ions produced by the alternative reactant ions, regardless of whether nitrogen oxide or dioxide was applied, is much higher. Despite the lack of measurements for comparable concentrations of admixtures, it can be seen that doping with nitrogen dioxide may be less effective.

Interesting conclusions can be drawn based on the set of calibration curves plotted for TDI measurements carried out at different temperatures (Fig. 6c, d). Irrespective of whether the dopant is used, a better sensitivity of the measurements of TDI carried out at higher temperatures is observed. For the temperature of 120 °C, the addition of nitric oxide dopant only slightly increases the IMS detector signal. At lower operating temperatures of the detector, when nitrogen oxide is applied, the increase of sensitivity is observed in comparison to the measurements conducted with pure carrier gas. However, the beneficial effect of dopant decreases with increasing temperature. Whereas a significant improvement in sensitivity when using a pure carrier gas, which is observed at higher temperatures, can be caused by lower hydration of hydronium ions at higher temperatures. This happens because the phenomenon of attaching the subsequent water molecules to hydronium ion clusters is observed at lower temperatures [35, 36]. Moreover, the proton affinity of water molecules is 691 kJ/mol [37]; however, hydronium ions available in the IMS detector are in the form of hydrated clusters [36], and the effective proton affinity of these ions cores is much higher. In order to calculate its value, it is necessary to add the enthalpies of subsequent attachments of water molecules to the PA of neutral molecules [34, 37, 38]. In effect, the PA of hydronium ions cores at lower temperatures is higher, and therefore, the proton transfer reaction is possible only in the case of analytes with a very high PA. On the contrary, at higher temperatures, when the reactant ions are less hydrated and the corresponding PA of neutral molecules is lower, the ionization via proton transfer reaction is also possible for analytes with lower values of proton affinity. This effect was observed also for other chosen analytes, however in smaller scale than in the case of TDI.

The calibration curves plotted based on results of measurements of DMMP (Fig. 6e) confirm the observation that the use of nitrogen oxides as dopants to the carrier gas in IMS for the detection of compounds with relatively high PA, like organophosphorus compounds, does not affect the efficiency of the ionization of this type of analyte, and thus their detection with ion mobility spectrometry. Very similar results were obtained for the measurements of TEP and NO_2_ dopant.

**Fig. 7:**
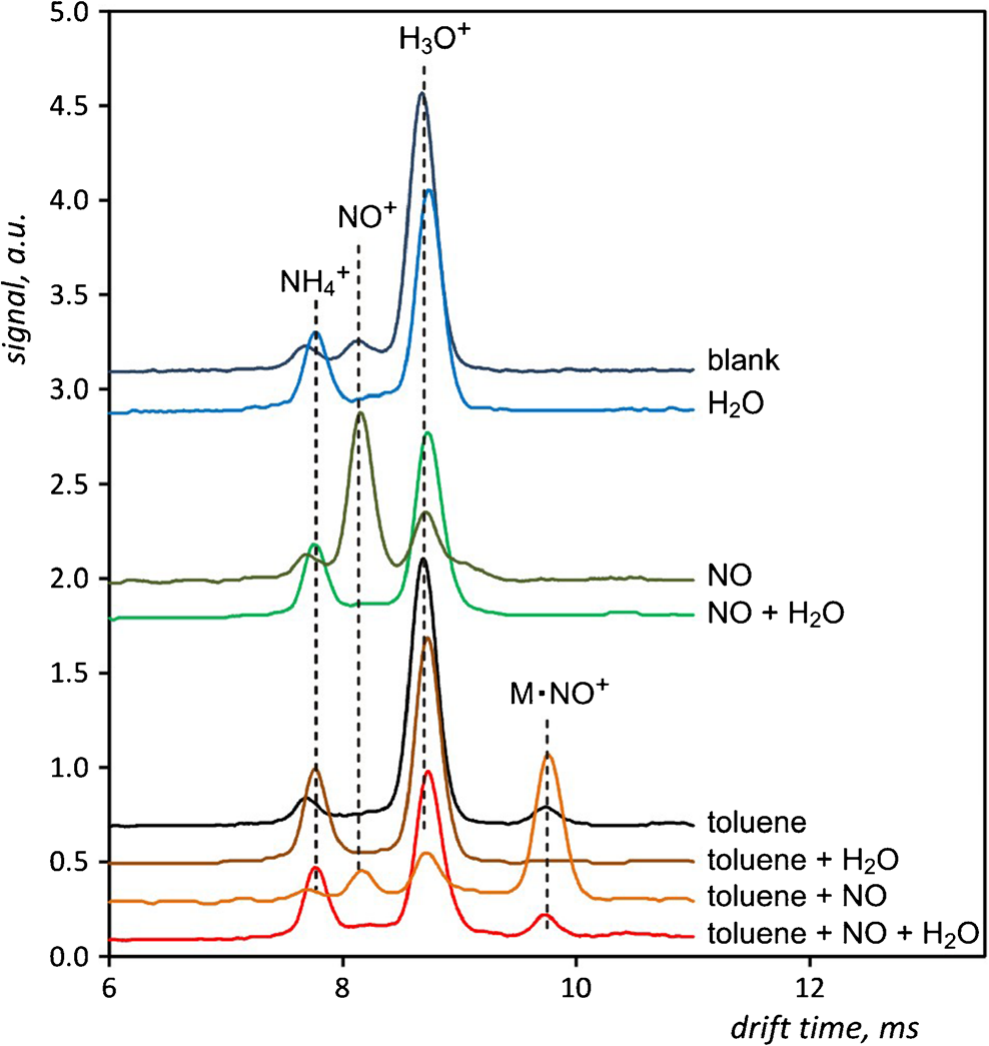
Drift time spectra registered for studies of influence of humidity on detection with IMS carried out with use of NO at 80 °C

To evaluate the effect of humidity on the effectiveness of doping with nitrogen oxide, the study in which the mixed gas introduced into the detector contained the analyte, NO admixture and water vapour was conducted. Measurements were carried out at 80 °C and did not have the quantitative character, but the concentrations of all substances in the respective measurements were maintained at around 12 ppm for NO, 20 ppm for toluene and 250 ppm for water vapour. Comparison of the drift times spectra recorded for these measurements is given in Fig. 7. The introduction of water vapour immediately suppresses the NO^+^ peak in the drift time spectrum, irrespective of whether nitrogen oxides were contained in the pure carrier gas or introduced from the cylinder. This disturbs the detection of toluene in the tests carried out without the use of a dopant. Its addition, however, although the water vapour is present in the carrier gas, enabled the detection of the analyte. The corresponding peak in the drift time spectrum is much smaller than in measurements carried out without water addition. The results are promising and will form the basis of further research conducted by the authors.

## Conclusion

The application of NO_x_ admixtures to the carrier gas in IMS is interesting from an analytical point of view. The use of these dopants in positive mode can significantly increase the sensitivity of the measurements which has great importance, especially in the case of aromatic compounds analyses. The sensitivity enhancement effect is observed for those compounds which are not easily ionized by proton transfer reactions, i.e., in case of this research, benzene, toluene and TDI. In higher temperatures, the ionization processes, with use of both, nitrogen oxides ions and standard hydronium ions, are more effective, and thus, the increase of sensitivity is achieved. Experiments with dopants introduced from the gas cylinder into the carrier gas showed that the results obtained with the addition of NO are similar to those obtained using NO_2_. The reactions of ionization with NO^+^ ions are very well described in many publications. In the case of NO_2_^+^, there is little information available in literature on their participation in ion-molecule reactions. The results of our research could not explain the course of ionization with NO_2_^+^; therefore, there is a need of carrying out more profound investigations. It is very well known and generally observed behaviour that the presence of water vapour significantly decreases the sensitivity. However, the use of nitrogen oxides as admixtures to the carrier gas in IMS allows the observed effect of humidity to be reduced.

The presence of nitrogen oxides does not affect the detection of substances characterized by relatively high PA. It was shown for organophosphorus compounds such as DMMP or TEP.

The use of these dopants may pose a risk related to introducing nitrogen oxides into the instrument; however, this destructive effect has not been investigated.
